# Editorial

**Published:** 1872-10-15

**Authors:** Aug. Rhoads

**Affiliations:** Papinville, Missouri


MEDICAL EXAMINER.

*A Semi-Monthly Journal of Medical Sciences.*

EDITED BY

N. S. DAVIS, M. D., AND F. H. DAVIS, M. D.

Chicago, October 15th, 1872.

### EDITORIAL.

PAPINVILLE, MISSOURI.

*Prof. N. S. Davis, Chicago, Ill.*

DEAR SIR:—I have thought often that I would write to you concerning a disease that has troubled me much for years; but I felt as if I would be imposing on your overworked time, and have postponed until now. The disease I wish to speak about, is commonly called Nursing Sore Mouth. I have met with about ten cases in six years, and within two years I have treated five cases. I have consulted all the authorities I could get hold of, and have been able to get little or no information from them; a great many of the works say nothing about it.

The disease, according to my experience, makes its appearance, in a majority of cases, about ten days after confinement—though it does come occasionally during the latter weeks of pregnancy. The symptoms are about as follows: The patient complains of loss of appetite, becomes pale, and nervous, and within a few days will complain of a rawness of the tongue, with smarting sensation when anything warm is taken into the mouth. On examination we find the tongue very red, as if it had been scalded, and in a few days the mucous membrane begins to peel off in strips or patches, and the tongue begins to look better. The disease appears now to settle down to a few ulcers on the back part and edges, with two or three elevations that look like warts. Matters continue about this way, so far as the tongue and mouth is concerned; the appetite continues to be poor; there is gradual emaciation. All these symptoms continue for a month or two. If not stopped, the œsophagus and upper portion of the stomach becomes involved, after which, in some cases, gradual emaciation continues until death closes the scene. I have seen two cases where there was every evidence of extensive ulceration of the stomach, accompanied with an anemic condition of the blood, that tonics had no control over, so long as the child nursed. From what I have seen of the disease, I am compelled to think that it is at first sympathetic with the secretions of the breast, for in every case where I have seen it tried, as soon as the child is taken from the breast, the mouth and tongue gets well, and remains so until a subsequent confinement, when it invariably returns.

I will now speak of treatment. I have tried almost everything that seemed to be suitable, for such a condition of the system, with little or no benefit. Iodide potass. and other altertives have no control over it. Iron and other tonics do but little good. Local applications frequently agravate the disease. A solution of tannic acid is the best local treatment I have found. Carbolic acid does more harm than good. The only thing that comes near being a cure, in my experience, is weaning the child.

Is there no remedy known to yourself for the above disease? if there is, you will confer a lasting benefit by letting it be known through the *Medical Examiner*, and perhaps save many lives. Yours, truly,

AUG. RHOADS, M. D.

NOTE.—The above letter was received many months since, and marked for editorial notice. But evidently it got covered by other papers, and escaped further attention until the present time. In reply to the inquiries of our correspondent, we would say, that we know of no specific cure for Nursing Sore Mouth. And yet it is more than fifteen years since a case has originated in the circle of our own practice, in which it became necessary to wean the child. Many years since, a careful study of the phenomena of the disease, led me to regard it as arising essentially from a deficiency of phosphatic salts in the blood. The woman, in furnishing from the blood the material necessary for the nutrition and growth of herself and child, does not assimilate these salts fast enough to supply the demand. The indication for treatment is chiefly to supply this deficiency; and no *tonics*, merely as such, which do not contain these salts as prominent constituents, will do any permanent good. If, as soon as the patient begins to feel the scalding and tender sensations in the edges of the tongue and mouth, she is required to take regularly a fluid drachm of the compound syrup of the hypophosphites at each meal-time, and at bed-time, with plain, nutritious food, and a little fresh, out-of-door air, every day, it will generally arrest the progress of the disease effectually, and she will remain well so long as she continues the medicine from two to four times a day. But in many instances it will be found necessary to keep up the supply of medicine during the whole period of nursing.

In several instances under our care, the omission of the medicine for two weeks during any part of the time would result in a renewal of the symptoms of trouble in the mouth and stomach. But with the renewal of the medicine the symptoms would disappear.

There are several other prescriptions which will answer a similar purpose. One of the best, is the mixture of extract of malt, two parts, and compound syrup of hypophosphites, one part, given in doses of a small table-spoonful at at each meal time. If the disease is neglected, or treated with ordinary tonics, or stimulants, or both, until the mucous membrane of the mouth, fauces, stomach, etc., is already extensively ulcerated, and the blood of the patient extremely impoverished, no treatment may supercede the necessity for weaning the child.

